# Evaluation of a local anaesthetic biopsy service for suspected cancers at a tertiary head and neck unit: relevance to post-COVID-19 recovery of surgical services

**DOI:** 10.1308/rcsann.2025.0027

**Published:** 2025-06-11

**Authors:** NN Vakharia, RC Dwivedi

**Affiliations:** ^1^ University College London Hospitals NHS Foundation Trust, UK; ^2^ NHS Grampian, UK; ^3^ University College London, UK

**Keywords:** Head and neck cancer, Biopsy, Local anaesthesia, Transnasal endoscopy

## Abstract

**Introduction:**

In response to pressures from the COVID-19 pandemic, a local anaesthetic (LA) biopsy service for patients with suspected head and neck cancer was set up at our centre.

**Methods:**

This study was a prospective audit of patients referred for LA biopsy of head and neck lesions over a 2-year period at an adult United Kingdom tertiary head and neck centre.

**Results:**

In total, 202 patients had LA biopsy during the audit period. Most common types of biopsies were transoral (*n* = 65, 32.3%) and transnasal endoscopy and biopsy (*n* = 59, 29.2%). Some 72.8% (*n* = 147) of lesions were benign, whereas 25.7% (*n* = 52) of lesions were malignant. One specimen did not arrive at the laboratory and two specimens did not survive transportation/processing, necessitating repeat biopsies. Five patients required repeat biopsy following initial non-malignant histology result (2.47%), three of which required biopsy performed under general anaesthetic (1.49%). There were no identified post-procedure complications.

**Conclusions:**

LA biopsy including transnasal oesophagoscopy/endoscopy is safe, well tolerated and can be used to assess patients with suspected head and neck cancer. Advantages include avoiding the risks of general anaesthesia and freeing up theatre capacity for more complex cases. We estimate savings of £900,000 over 2 years. Faced with limited theatre capacity and growing waiting lists, LA biopsy can also improve time to diagnosis and treatment for head and neck malignancies. We demonstrate the benefits of LA biopsy and highlight the role of transnasal oesophagoscopy/endoscopy in the recovery of surgical services in otolaryngology departments across the world in the post-pandemic era.

## Introduction

Traditionally, procedures such as panendoscopy under general anaesthesia (GA) are performed for the diagnosis and management of head and neck (H&N) conditions.^1^ With increasing life expectancy and improving management of chronic medical conditions, GA in older patients is increasingly performed, but involves thorough preoperative assessment, which is resource intensive and introduces delay to diagnosis.^2^ Management of H&N malignancies often requires two stages. First, examination and biopsy to assess extent of disease and obtain histological confirmation. Second, based on the clinical, radiological and histological findings, patients may require further procedures for resection of the lesion. Some patients may therefore require GA on multiple occasions, with the accompanying risks of each.

Examination and biopsy of H&N lesions, including upper aerodigestive tract (UADT) lesions under local anaesthesia (LA) has been performed for many years, with indirect laryngoscopy dating back to the 19th century.^3,^^4^ The combination of indirect visualisation via the oral cavity and the use of curved transoral instruments makes biopsy of hypopharyngeal and laryngeal lesions technically difficult to perform and poorly tolerated by patients,^5^ often requiring sedation.^6^

Technological advances in ‘chip-in-tip’ flexible endoscopes have resulted in improved image quality and visualisation of suspected UADT lesions. Reduction in the calibre of flexible endoscopes and integration of both suction and 2.4mm working channels allows the passage of channelled endoscopes and biopsy via the nasal passage for UADT lesions. Transnasal oesophagoscopy/endoscopy (TNO/TNE) allows examination and instrumentation from the nasopharynx to the trachea and distal oesophagus under LA, typically in a clinic or procedure room setting because sedation or GA is not required.^1,7,8^ TNE also avoids the risk of trauma to lips and teeth associated with traditional panendoscopy.^9^

Aside from use of TNO/TNE, other H&N lesions detected during clinical evaluation are often amenable to LA biopsy, such as postnasal space lesions via rigid nasendoscopy, or excision of enlarged lymph nodes.

By performing biopsy under LA, preoperative assessment and GA can be avoided, reducing time to diagnosis and commencement of treatment, while freeing up GA theatre capacity for advanced and complex procedures.

During the COVID-19 pandemic and post-COVID-19 period, surgical specialties have faced unprecedented demand.^10^ Growing waiting lists and a restriction of theatre capacity have resulted in delay in diagnosis, including in those with H&N conditions. Early diagnosis and treatment are associated with improved outcomes in H&N malignancies, with delays in diagnosis and intervention detrimental to prognosis.^11^ Although post-COVID-19 recovery strategies have been implemented across all specialties, shortages in theatre capacity and staffing persist.^12^ Safe diversion of procedures away from GA operating theatres can provide much needed respite to strained services.

At our tertiary H&N centre, in response to mounting pressures on theatre availability and rising waiting times for cases of potential H&N cancer in adults during the COVID-19 pandemic, we set up an LA biopsy service in June 2020. Based in our outpatient department, the service commenced under the supervision of the senior author. The objective was to safely divert cases away from the operating theatre and provide timely diagnosis for suspected cancer patients, simultaneously freeing up valuable GA theatre space for those awaiting more complex surgeries. The service has continued during the post-COVID-19 recovery period to date. Here, we present the results from an audit of the first 2 years of this service, demonstrating the advantages of an LA biopsy service and its role in the recovery of surgical services following the COVID-19 pandemic.

## Methods

Patients referred via the suspected cancer referral pathway are assessed in a face-to-face outpatient appointment. Following clinical evaluation, patients with potentially malignant UADT lesions are offered biopsy under LA if this is felt to be suitable and safe to perform outside the operating theatre. All procedures are performed in a dedicated procedure room in the ear, nose and throat (ENT) outpatient building after acquiring informed consent. A pulse oximeter is applied to monitor oxygen saturation and heart rate during the procedure.

### Transnasal endoscopy and biopsy

For TNE, topical anaesthesia consists of lidocaine 5% with phenylephrine 0.5% spray, two sprays to each nasal passage, followed by lidocaine 10% spray, two sprays each to the nasal passages and oropharynx. Up to 5ml of lidocaine 4% is applied to the larynx via a mucosal atomisation device (MADgic laryngo-tracheal; Teleflex Medical Europe Ltd, Athlone, Republic of Ireland). Once LA has taken affect (approximately 5min), a transnasal oesophagoscope (Pentax, Tokyo, Japan) is passed via the nasal passage and the lesion is identified. Single-use 2mm flexible biopsy forceps are then passed by an assistant, via the working channel on the endoscope and multiple biopsies of the lesion are taken until a sufficient specimen has been obtained. In our experience, there is minimal bleeding post biopsy, and no specific haemostasis is required.

For nasal or nasopharyngeal lesions, topical anaesthesia to the oral cavity, oropharynx and supraglottis described above is not required. For anterior nasal lesions, LA is infiltrated (lidocaine 2% with adrenaline 1:80,000) and the lesion is excised under direct vision. For lesions deeper in the nasal passage, such as nasal polyps or postnasal space lesions, topical anaesthesia is applied to the nasal passages as described previously, a 0° rigid nasendoscope is passed to visualise the lesion and biopsies are taken using Blakesley forceps. Haemostasis is achieved with topical adrenaline, or bipolar cautery for anterior lesions.

### Transoral biopsy

For transoral biopsies of oropharyngeal or oral cavity lesions, lidocaine 10% spray is applied to the oral cavity and oropharynx to provide anaesthesia and suppress the gag reflex. For larger lesions, or those in the tongue base or tonsil, lidocaine 2% with adrenaline 1:80,000 is also infiltrated. Small lesions are excised with sharp dissection, whereas for larger lesions, a wedge biopsy is taken. Haemostasis is achieved with topical adrenaline and bipolar cautery.

### Skin lesion and lymph node excision

Excision of skin lesions or lymph nodes is performed following a standard technique, with skin preparation followed by infiltration of LA (lidocaine 2% with adrenaline 1:80,000). The lesion is excised, and haemostasis is achieved using bipolar cautery. Wound closure is performed with dissolvable or non-dissolvable sutures as required.

After all procedures, patients are observed for 30min and discharged if their observations are satisfactory. Patients who receive topical anaesthesia to the oropharynx and larynx are advised to remain nil-by-mouth for 60min post procedure due to the risk of inadvertent aspiration.

Following approval from our in-hospital audit board, data for patients managed in the LA biopsy service were prospectively collected in an encrypted database, including demographic data, patient comorbidities, final diagnosis and further management (surgical, oncological, palliative or none). Using comorbidity data, the Charlson Comorbidity Index (CCI) score was calculated for each patient. Patient electronic records were also reviewed to assess for any post-procedure complications. The AGREE reporting guidelines have been followed during this study.

## Results

From June 2020 to June 2022, 202 patients had a procedure performed under LA. The majority were male (58.4%), and the average age was 54.9 (range 17–92 years). Eight patients had CCI score of 7 or more, corresponding to a 0% 10-year survival rate (Table 1). The mean CCI score was 2.26, corresponding to a 10-year survival rate of between 77% and 90%.

**Table 1:** Charlson Comorbidity Index (CCI) for patients managed in the local anaesthetic biopsy service

CCI score	No. of patients	Estimated 10-year survival rate (%)
0	60	98
1	31	96
2	31	90
3	24	77
4	22	53
5	17	21
6	9	2
7	3	0
8	3	0
9	1	0
10	1	0
>10	0	0

The most common types of procedure were transoral biopsy (*n* = 65, 32.3%), and TNE and biopsy (*n* = 59, 29.2%). Biopsy of posterior nasal or nasopharyngeal lesions using a rigid endoscope accounted for 13.4% of cases, and 12.9% of procedures involved direct excision/biopsy of anterior nasal lesions.

Table 2 contains the breakdown of site and subsite of lesions biopsied. The most frequently biopsied region was the oropharynx (*n* = 69, 34.2%), followed by the larynx (*n* = 41, 20.3%), nasal cavity and sinuses (*n* = 31, 15.3%) and nasopharynx (*n* = 21, 10.4%). Further division into anatomical subsite is displayed in Table 2; the most common subsites were tonsil (18.8%), glottic (12.9%) and nasal cavity (12.9%).

**Table 2:** Site and subsite of lesions biopsied

Site	Subsite	*n*	%
**Oropharynx**		**69**	**34.2**
	Tonsil	38	18.8
	Base of tongue	9	4.5
	Soft palate	12	5.9
	Oropharyngeal wall	10	5.0
**Larynx**		**41**	**20.3**
	Supraglottic	14	6.9
	Glottic	26	12.9
	Subglottic	1	0.5
**Nasal cavity and sinuses**		**31**	**15.3**
	Nasal cavity	26	12.9
	Sinus	5	2.5
**Nasopharynx**		**21**	**10.4**
**Skin**		**17**	**8.4**
	Pinna	1	0.5
	Neck	10	5.0
	Temple	1	0.5
	Scalp	1	0.5
	Post auricular	2	1.0
	Preauricular	1	0.5
	Occiput	1	0.5
**Other**		**5**	**2.5**
	External auditory canal	4	2.0
	Laryngectomy stoma	1	0.5
**Hypopharynx**		**6**	**3.0**
	Pyriform sinuses	5	2.5
	Post-cricoid	0	0.0
	Posterior hypopharyngeal wall	1	0.5
**Cervical lymph node**		**6**	**3.0**
	Occipital chain	1	0.5
	Level 5	5	2.0
**Salivary gland**		**3**	**1.5**
	Submandibular gland	2	1.0
	Parotid gland	1	0.5
**Oral cavity**		**3**	**1.5**
	Tongue	1	0.5
	Lip	1	0.5
	Floor of mouth	1	0.5
**Total of lesions at each site**		**202**	**100**

Following histopathological analysis, 72.8% of lesions were benign (*n* = 147), and 25.7% of lesions were malignant (*n* = 52). One specimen did not arrive at the laboratory and two specimens did not survive transportation/processing for histology; these patients required repeat biopsy. Owing to clinical suspicion, five patients required a repeat biopsy following an initial non-malignant histology result (2.47%), three of whom required biopsy performed under GA (1.49%). Following discussion in the H&N multidisciplinary meeting, 30 patients (14.9%) were referred for oncological management, 21 patients (10.4%) were managed surgically and 4 patients (2.0%) were referred for palliative care. Some 68.8% of patients required no further management and were downgraded from the urgent suspected cancer pathway or discharged from H&N follow-up. There were no documented unscheduled reviews or attendances to the emergency department with post-procedure complications.

## Discussion

Our results demonstrate the value of a LA biopsy service to safely and accurately assess patients with H&N lesions. Procedures were performed on 202 patients across a wide age range and with various comorbidities, with no identified complications. Patients with multiple comorbidities would have required thorough anaesthetic preassessment to determine the safety of GA, but all patients were able to undergo LA biopsy, including those with a high CCI. Only three patients (1.49%) required repeat biopsy under GA.

Liaison with local pathology services is crucial, because the specimen size is smaller than those obtained during GA biopsy. Despite obtaining small tissue specimens, we achieved tissue diagnosis at the first attempt for all but three patients (199 patients, 98.5%). Clinical suspicion is required for lesions that appear malignant, with repeat biopsy performed if a result of dysplasia or carcinoma in situ is reported. Rates of 98.5% for single biopsy diagnosis and 25.7% for malignant findings are both higher than would be expected from traditional methods of biopsy, but we attribute this to the preselection of patients that would be amenable to LA biopsy and the resulting high pretest probability.

Thirty patients were referred for oncological management and four patients were managed with palliative care; thus GA was not required during their diagnostic process. Each of the 21 patients managed surgically required 1 fewer GA procedure, because diagnosis was made via LA biopsy. These patient groups benefitted from improved efficiency in time to diagnosis and reduced risk from GA.

While health services recover from the COVID-19 pandemic, efforts are required to improve efficiency and utilisation of limited theatre capacity. NHS England published a delivery plan for tackling the backlog, focusing on increasing capacity, prioritising treatment and transforming delivery of elective care. Independent sector facilities have been utilised both before and during the pandemic and have been highlighted as a means to increase capacity, but this is costly, with an estimated £2 billion spent by NHS England for independent sector utilisation during the pandemic.^13,^^14^ Prioritisation based on clinical urgency has also been utilised both prior to and during the pandemic, especially in cancer cases. Despite this, the number of patients with suspected cancer treated within 62 days from referral remains below the NHS England target of 85%.^15^ Furthermore, the Royal College of Surgeons of England’s ‘Advancing the Surgical Workforce: 2023 UK Surgical Workforce Census Report’ highlighted the ongoing scarcity in operating theatre time compared with pre-COVID-19 levels.^16^ The report found that more than half of the respondents felt limited access to theatre was one of the main challenges facing surgery and identified that 41% of consultants undertook just two scheduled operating sessions per week.

Methods for transforming the delivery of elective care include expansion of community diagnostic centres and surgical hubs. Low-complexity, safe procedures such as the LA biopsies described would lend themselves to community diagnostic centres. Provision of high-volume, low-complexity surgery at surgical hubs has been recommended by the Royal College of Surgeons of England to ease waiting list pressures through increased capacity.^17^ However, by diverting suitable diagnostic procedures to a safe, accurate LA service, further pressure can be taken off theatres, improving diagnostic and treatment targets for cancer patients.

After initial set-up costs, per procedure, LA biopsy services have consistently been shown to be significantly cheaper than GA biopsies, because anaesthetic preassessment, theatre space and sterilisation of theatre sets are not required.^18^ Healthcare staff requirements are fewer for LA procedures because an anaesthetist, operating department practitioner and postoperative nursing staff are not required.

Although H&N biopsy via TNO/TNE has been available for more than a decade, with positive results and safety of procedures demonstrated in studies outside the United Kingdom (UK), utilisation of TNO/TNE is limited in the UK.^19–21^ Reasons for the slow uptake include set-up costs and training requirements. In addition, traditional rigid endoscopy and direct visualisation of a tumour allow assessment of the extent of disease and suitability for resection. However, improved visualisation using TNE and improving technology in cross-sectional imaging allow surgeons to assess the possibility of surgical resection without GA examination. Patients managed with surgical resection of oropharyngeal or laryngeal malignancies had examination under anaesthesia as part of their resection to confirm the macroscopic extent of disease and ensure that resectability and planned closure (either directly or with the use of a flap if required) was appropriate. This included seven patients who had laser excision of laryngeal tumour, three patients who had laryngectomy with selective neck dissection, two patients who had transoral resection of oropharyngeal tumour and one total rhinectomy.

Based on an estimation of 1h per biopsy under GA, our LA service has saved 200h of GA theatre time, freeing up capacity for other cases. Although there are no clear data on the cost of running a GA theatre, the ‘Improving quality and efficiency in the operating theatre’ report (2009) estimated an average of £1,200 per hour.^22^ Adjusting for inflation, this equates to approximately £1,985 per hour today. Extra theatre capacity can be utilised for more complex procedures, attracting a higher national tariff system payment than GA biopsy. Using data from the 2022/2023 NHS National Tariff Payment system, the price of diagnostic laryngoscopy or pharyngoscopy is £150, whether this is performed in the outpatient clinic or electively under GA.^23^ In comparison, the price of thyroid procedures range from £3,333 to £4,753 based on patient comorbidity, and H&N procedures range from £1,653 for minor procedures to £17,916 for complex procedures in comorbid patients. Utilising theatre capacity for complex procedures will, therefore, result in higher revenue.

LA biopsy patients do not require preanaesthetic workup, which can require further costly investigations such as echocardiography or imaging. National Institute for Health and Care Excellence guideline NG45 details the costs of common tests required during preoperative assessment, such as bloods tests, urinalysis and pulmonary functions tests, along with additional costs of follow-up investigations triggered by initial tests.^24^ Although these vary significantly between patients, estimated costs of up to £1,110.00 (adjusted for inflation) could be incurred for patients with complex comorbidities. In addition, post-anaesthesia recovery, with one-to-one nursing and other consumable resources, is not required following LA biopsy. Further costs may be incurred following GA if a patient requires overnight admission, with an estimated cost of £586.59 per bed day in 2016 or £788.00 with inflation.^25^

Although an accurate value is difficult to calculate, if we transcribe these estimated savings into financial terms, we conservatively estimate that our LA procedure service has saved £4,500.00 per patient, equating to £900,000 over 2 years. If similar services are offered across the majority of ENT and H&N services, there is potential for significant financial and resource savings for an already strained NHS.

To set up a similar service based on our model, collaboration is required from numerous departments, including ENT to provide surgical staff, theatres to supply a scrub and circulating nurse, the outpatient department to provide a space for procedures to be performed and nursing staff to care for patients pre- and post-biopsy. In addition, the pathology department will need to be informed regarding the relatively small specimen sizes, and the sterile services and decontamination department will need to be able to provide appropriate decontamination of equipment. Given the existing evidence in the literature regarding the safety and accuracy of LA biopsies, there were no legal concerns when setting up our service, but the availability of emergency resuscitation equipment is essential. Our service was set up as an outpatient biopsy clinic with each procedure initially allocated to a 1h-long outpatient appointment. Once established, we found that 45min appointments were sufficient.

## Conclusions

The impact of the COVID-19 pandemic on surgical services across the globe is likely to last for many years. A change in approach from performing biopsies under GA can help reduce strain, diverting patients away from operating theatres, reducing resource requirements and time to diagnosis. We have demonstrated that an LA biopsy service for patients with suspected H&N malignancy can be safely delivered, including in patients with multiple comorbidities who may be unfit for GA. Furthermore, our LA biopsy service model is ideally suited to the delivery plan for tackling the elective care backlog published by NHS England. Similar results and benefits have been demonstrated by gastroenterologists for assessment of the upper gastrointestinal tract, with TNO/TNE in the outpatient setting, seen as a crucial tool for the recovery of diagnostic services.^26^ Although initial set-up costs, training and a change from tradition are required, the potential benefits of rolling out LA biopsy services are clear to see, with significant financial savings, improved diagnostic efficiency and replicability throughout the world.
